# Risk factors for and outcomes of heatstroke-related intracerebral hemorrhage

**DOI:** 10.1097/MD.0000000000037739

**Published:** 2024-04-19

**Authors:** Guodong Lin, Chongxiao Xu, Jieyi Wu, Hailun Peng, Anwei Liu, Xuan He, Wenda Chen, Xiaogan Hou, Qiang Wen, Zhiguo Pan

**Affiliations:** a Department of Critical Care Medicine, General Hospital of Southern Theatre Command of PLA, Guangzhou, China; b The First School of Clinical Medicine, Southern Medical University, Guangzhou, Guangdong, China; c Department of Anesthesiology, General Hospital of Southern Theatre Command of PLA, Guangzhou, China.

**Keywords:** brain injury, disseminated intravascular coagulation, heatstroke, intracerebral hemorrhage

## Abstract

Some patients with heatstroke also experience intracerebral hemorrhage (ICH). However, clinical case reports of heatstroke-induced ICH are rare. The risk factors for cerebral hemorrhage after heatstroke remain unknown. The present study evaluated the clinical characteristics and risk factors of patients with heatstroke-related ICH. In this retrospective observational study, we collected data on all ICHs after heatstroke occurred between 2012 and 2022. The characteristics of patients with heatstroke-induced ICH were described. The risk factors for cerebral hemorrhage after heatstroke were examined using logistic regression analysis. In total, 177 patients were included in this study, and 11 patients with ICH secondary to heatstroke were identified. Variables with *P* values of <.05 in univariate models, comparing the cerebral hemorrhage and control groups, included heatstroke cause, temperature, heart rate, respiratory rate, vasopressor use, mechanical ventilation use, Acute Physiology and Chronic Health Evaluation II, total bilirubin, creatinine, platelet count, prothrombin time, procalcitonin, creatine kinase, disseminated intravascular coagulation (DIC) occurrence, and DIC score. Multivariate logistic regression showed that heatstroke patients with higher DIC scores (odds ratio, 18.402, 95% confidence interval, 1.384–244.763, *P* = .027) and higher creatine kinase levels (odds ratio, 1.021, 95% confidence interval, 1.002–1.041, *P* = .033) were at a higher risk of developing ICH. The death rate was higher in the cerebral hemorrhage group than in the control group (*P* = .042). Heatstroke-related cerebral hemorrhage may be associated with elevated creatinine levels and DIC severity (International Society on Thrombosis and Hemostasis score) after heatstroke, and heatstroke with cerebral hemorrhage may accelerate death.

## 1. Introduction

Severe heatstroke is defined as a disease in which the central body temperature rises above 40°C, causing central nervous system (CNS) disturbances, resulting in delirium, convulsions, or coma, after exposure to a high-heat environment (classic type) or strenuous physical labor (exertional type).^[1–3]^ Severe heatstroke is often combined with multiple organ dysfunction and damage to the coagulation system, striated muscles, liver, CNS, lungs, kidneys, intestines, and other vital organs.^[3–5]^ When associated with multiple organ failure, heatstroke is associated with an increased mortality rate.^[2,5,6]^ The initiation of disseminated intravascular coagulation (DIC) is an important factor for the aggravation of multiple organ failure in severe heatstroke.^[4,5,7,8]^ Severe DIC can lead to systemic hemorrhage, with cerebral hemorrhage being the most serious manifestation. Once cerebral hemorrhage occurs, the patient’s prognosis may be poor. Currently, there are sporadic reports of cerebral hemorrhage secondary to heatstroke.^[4,9]^ In the present retrospective clinical study, we explored the clinical characteristics and risk factors of heatstroke-related intracerebral hemorrhage (ICH).

## 2. Methods

### 2.1. Study population, setting, and data collection

This was a retrospective single-center observational study and included patients with heatstroke admitted to the intensive care unit (ICU) at the General Hospital of Southern Theater Command of PLA from 2012 to 2022. The inclusion criteria for the study were patients presenting with a heatstroke. The exclusion criteria were patients aged ˂15 years, patients with incomplete data, and patients who abandoned treatment and were discharged from the hospital. The included patients were divided into 2 groups (cerebral hemorrhage group and a control group). Patients’ clinical data were collected through the hospital’s case system. Data on demographic characteristics, clinical signs and symptoms at presentation, and laboratory and radiologic test results obtained during the ICU admission were obtained. Written informed consent was obtained from all study participants or their families on their behalf. This retrospective study design was approved by the institutional ethics review board.

### 2.2. Study definitions

The clinical diagnosis of heatstroke was based on a triad of CNS alteration, hyperthermia, and a history of exposure to extreme environmental heat or vigorous muscle exertion. Cerebral hemorrhage was diagnosed based on computed tomography scan results.

### 2.3. Statistical analyses

Stata 15.1 software (Stata Corp. LLC, College Station, TX) was used for statistical analysis. The Shapiro–Wilk test was used to test the normal distribution of numerical variables. Continuous variables are expressed as means with standard deviations or medians with interquartile ranges if the data are not normally distributed. Categorical data are expressed as counts and percentages. The Wilcoxon rank-sum test was used to compare non-normally distributed continuous variables between the 2 groups. The Chi-square test was used for contingency tables, and Fisher exact test was used to analyze contingency tables with small sample sizes. Logistic regression analysis was performed on selected independent variables. Results were considered significant at *P* values of <.05.

## 3. Results

### 3.1. Patient demographic and clinical characteristics

**Figure 1.**
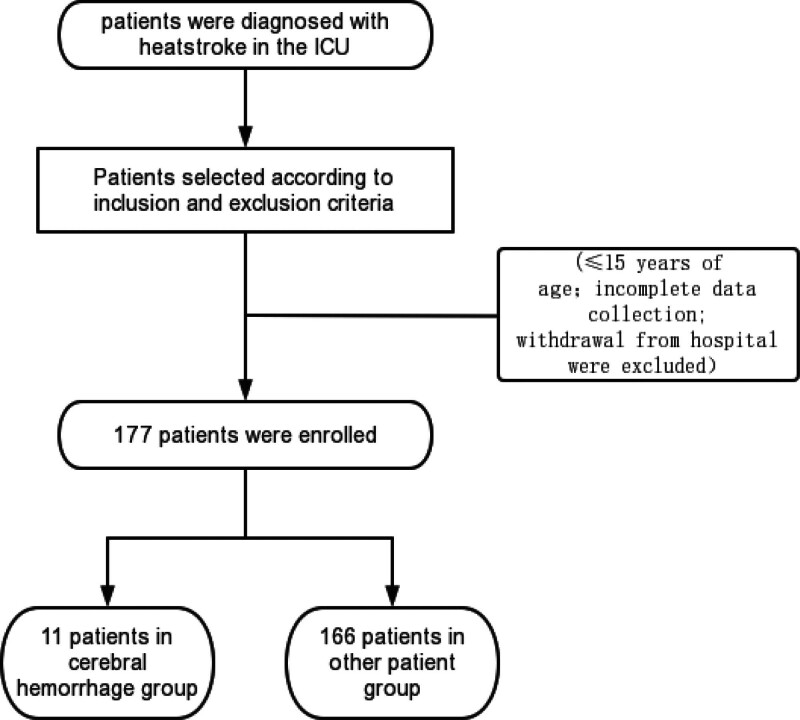
Study flow diagram. ICU = intensive care unit.

In total, 177 patients were included in this study after eligibility screening. Eleven cases of cerebral hemorrhage following a heatstroke were included (Fig. 1). All patients were male, with a median age of 21 years. In total, 173 (97.74%) patients experienced an exertional-type heatstroke, and 4 (2.26%) patients experienced a classic heatstroke. The body temperature of the patients in the cerebral hemorrhage group was 38 (37.4–38.9)°C, which was significantly higher than that of the control group (37 [36.5–37.8]°C). The heart rate (110 [99–127] bpm) of patients with cerebral hemorrhage was significantly higher than that of the controls (83 [70–100] bpm), and the respiratory rate of patients with cerebral hemorrhage was higher than that of controls. There was no significant difference in the mean arterial pressure at admission, but the patients in the cerebral hemorrhage group needed more vasoactive drugs (*P* = .029). Furthermore, 63.64% of patients with cerebral hemorrhage needed mechanical ventilation. The clinical characteristics of the patients are presented in Table 1. Finally, 81.8% of patients in the cerebral hemorrhage group had disturbance of consciousness, and their Glasgow Coma Scale (GCS) score was 6 (3–15) points. Compared with the control group, the patients showed a deeper coma (*P* = .0001).

**Table 1:** Baseline clinical features and outcomes of heatstroke patients with cerebral.

Characteristic	All patient (n = 177)	Patient with cerebral hemorrhage (n = 166)	Patient with cerebral hemorrhage (n = 11)	*P* value
Sex, n (%)				
Male (%)	176 (99.44)	165 (99.40)	11 (100)	.938
Age, yr	21 (19–26)	21 (19–26)	21 (18–27)	.6580
Heatstroke cause (%)				.02
Exertional	173 (97.74)	164 (98.80)	9 (81.82)	
Classical	4 (2.26)	2 (1.20)	2 (18.18)	
Temperature, °C	37.2 (36.5–37.9)	37 (36.5–37.8)	38 (37.4–38.9)	.0105
Heart rate, bpm	85 (71–103)	83 (70–100)	110 (99–127)	.0007
Respiratory rate, bpm	20 (20–20)	20 (20–20)	21 (20–24)	.0249
MAP, mm Hg	85.33 (77.33–93)	85.33 (77.33–93)	81.67 (65.33–94.67)	.8197
Vasopressor use, %	12 (6.78)	9 (5.42)	3 (2.73)	.029
Mechanical ventilation use, %	37 (20.90)	30 (18.07)	7 (63.64)	.002
GCS	15 (15–15)	15 (15–15)	6 (3–15)	.0001
APACHE II	3 (2–10)	3 (2–8)	20 (9–26)	.0000
TBIL, μmol/L	16.9 (11.8–35.4)	16.4 (11.6–33.9)	67.7 (23.3–103.6)	.0034
Cre, μmol/L	113 (83–153)	108 (81.5–145)	324 (206–345)	.0000
PLT, 10^9^/L	136 (80–181)	137.5 (84.5–185)	57.5 (29–136)	.0067
PT, s	17.1 (15.1–23)	17 (15.05–21.6)	33.9 (16.9–39.8)	.0221
WBC, 10^9^/L	11.495 (8.6–14.51)	11.495 (8.62–14.52)	11.13 (7.78–14.346)	.9897
PCT, ng/mL	1.745 (0.71–3.84)	1.6 (0.645–3.585)	4.75 (1.7–18.67)	.0137
CK, U/L	987 (405–2762)	891 (339–2757)	1710 (1272–5483)	.0280
DIC occurrence, %	50 (28.25)	41 (24.70)	9 (81.82)	.0000
DIC score	1 (0–5)	1 (0–4)	7 (6–8)	.0000
Outcome				
Mortality, %	25 (14.12)	15 (9..04)	10 (90.91)	.0000
Length of ICU stay	5 (3–12)	5 (3–11)	12 (7–18)	.0349
Length of hospital stay	11 (5–23)	11 (4–24)	12 (7–18)	.9565

APACHE II = Acute Physiology and Chronic Health Evaluation II, CK = creatine kinase, Cre = creatinine, DIC = disseminated intravascular coagulation, GCS = Glasgow Coma Scale, ICU = intensive care unit, MAP = Mean arterial pressure, PCT = procalcitonin, PLT = platelet count, PT = prothrombin time, TBIL = total bilirubin, WBC = White blood cells.

### 3.2. Organ function

The mean Acute Physiology and Chronic Health Evaluation II (APACHE II) score of all patients was 3 (2–10) points; the score of the cerebral hemorrhage group (20 [9–26]) was significantly higher than that of the control group (*P* = .000). Organ damage in the cerebral hemorrhage group was more extensive than that in the control group. The total bilirubin level in the cerebral hemorrhage group (67.7 [23.3–103.6] μmol/L) and the creatinine (Cre) level (324 [206–345] μmol/L) were significantly higher than those in the control group. The platelet level in the cerebral hemorrhage group was 57.5 (29–136) 10^9^/L, which was significantly lower than that in the control group (*P* = .000). The prothrombin time in the cerebral hemorrhage group (33.9 [16.9–39.8]) was significantly longer than that in the control group (*P* = .0221). In terms of infection indicators, procalcitonin (PCT) in the cerebral hemorrhage group was significantly higher than that in the control group (*P* = .0137); however, there was no significant difference in white blood cell counts. The rhabdomyolysis index, at approximately creatine kinase (CK) 1710 (1272–5483) U/L, was also significantly higher in the cerebral hemorrhage group than in the control group (*P* = .0280). Moreover, the incidence of DIC (90.91%) and the international society on thrombosis and hemostasis (ISTH) score in the cerebral hemorrhage group (7 [6–8] points) were significantly higher than those in the control group.

### 3.3. Risk factors for intracerebral hemorrhage in heatstroke patients

Variables with *P* values of <.05 in the univariate models included heatstroke cause, temperature, heart rate, respiratory rate, vasopressor use, mechanical ventilation use, GCS, APACHE II, total bilirubin, Cre, platelet count, prothrombin time, PCT, CK, DIC occurrence, and DIC score (Table 2). Clinically relevant variables were included in multivariate logistic regression models for ICH in patients with heatstroke. Patients with Heatstroke with higher DIC scores (odds ratio: 18.402, 95% confidence interval: 1.384–244.763, *P* = .027) and higher CK levels (odds ratio: 1.021, 95% confidence interval: 1.002–1.041, *P* = .033) were at a higher risk for developing ICH.

**Table 2:** Risk factors for cerebral hemorrhage in heatstroke patients (logistic regression).

Variables	Multivariate analysis	
	Adjusted OR (95% CI)	*P* value
Heatstroke cause	301.53 (0.000–393000)	.427
Temperature	11.50 (0.525–251.866)	.121
Heart rate	0.974 (0.889–1.067)	.567
Respiratory rate	1.197 (0.606–2.363)	.605
Vasopressor use	0.234 (0.000–111.157)	.644
Mechanical ventilation use	0.941 (0.002–445.248)	.985
APACHE II	1.042 (0.618–1.756)	.878
GCS	1.328 (0.582–3.026)	.500
TBIL	1.005 (0.962–1.050)	.830
Cre	1.021 (1.002–1.041)	.033
PLT	1.034 (0.985–1.085)	.177
PT	1.066 (0.910–1.249)	.431
PCT	1.056 (0.605–1.844)	.847
CK	1.000 (0.999–1.001)	.734
DIC occurrence	0.006 (0.000–7.742)	.160
DIC score	18.402 (1.384–244.763)	.027

APACHE II = Acute Physiology and Chronic Health Evaluation II, CI = confidence interval, CK = creatine kinase, Cre = creatinine, DIC = disseminated intravascular coagulation, GCS = Glasgow Coma Scale, OR = odds ratio, PCT = procalcitonin, PLT = platelet count, PT = prothrombin time, TBIL = total bilirubin.

### 3.4. Intracerebral hemorrhage

The sites of ICH were as follows: subarachnoid (63.6%), lobar (27.3%), cerebellar (18.2%), basal ganglia (9.1%), and Foramen ovale (9.1%). Cerebral hemorrhage occurred 5 (1–10) days after heatstroke.

### 3.5. Outcomes

The overall mortality rate of all patients was 14.12%, and the mortality rate of patients in the cerebral hemorrhage group was 90.91%, which was significantly higher than that of the control group (*P* = .000). Multivariate analysis (after adjusting for factors such as heatstroke cause, temperature, heart rate, vasopressor use, mechanical ventilation use, GCS, APACHE II, PCT, CK, DIC occurrence, and DIC score) showed that patients with cerebral hemorrhage had a higher mortality rate (*P* = .042) but similar ICU stay time (Table 3) as that of the control group. In the cerebral hemorrhage group, death occurred 4 (1–5) days after ICH.

**Table 3:** Multivariate analysis for outcomes of cerebral hemorrhage in heatstroke patients.

Outcomes	Multivariate analysis Adjusted OR (95% CI)	*P* value
Mortality	0.0000–0.7642	.042
Length of ICU stay	0.9148–1.0806	.892

CI = confidence interval, ICU = intensive care unit, OR = odds ratio.

## 4. Discussion

In total, 177 patients with heatstroke were included in this study, of which 11 had cerebral hemorrhage caused by severe heatstroke. The present study summarizes the characteristics of patients with cerebral hemorrhage and other patients with heatstroke. Compared with other patients, those in the cerebral hemorrhage group had higher body temperature, faster heart rate, higher respiratory rate, more vasoactive drug use, more ventilator support, deeper GCS, more severe disease, and more severe organ damage (liver, kidney, coagulation, infection). Multivariate analysis showed that the severity of renal insufficiency and DIC were risk factors for cerebral hemorrhage. The mortality in patients with heatstroke with cerebral hemorrhage tended to increase. The most common sites of hemorrhage were the subarachnoid, lobe, and cerebellum.

Brain injury after severe heatstroke is a common clinical manifestation.^[3,10–13]^ Early symptoms include behavioral changes, confusion, delirium, dizziness, weakness, agitation, combativeness, slurred speech, nausea, and vomiting.^[1,3]^ Seizures and sphincter incontinence may occur in severe cases, mainly in exertional heatstroke. These symptoms may manifest as a disturbed mental state, including confusion, delirium, or lethargy.^[1–3]^ Other neurological findings include seizures, severe alteration in the level of consciousness, including deep coma, areflexia, miosis, and absence of brainstem reflexes.^[2,3]^ The mechanisms underlying brain injury include direct thermal shock, endothelial cell damage, inflammatory responses, ischemia and hypoxia, activation of coagulation, and oxidative stress.^[3,6,10–12]^ Heatstroke-related cerebral hemorrhage is relatively rare.^[4,9]^ Thus far, only 2 cases have been reported, one of which was described by Boersma et al^[9]^ in 1998; a young, healthy male patient died due to exertional heatstroke, and the cause of the patient’s cerebral hemorrhage was summarized as coagulopathy in the setting of exertional heatstroke. Another case report, published in 2012 by Satyendra et al,^[4]^ described a 38-year-old male patient who presented at the emergency department with a history of sudden loss of consciousness during a 10-km run. The patient was diagnosed with heatstroke and DIC, had bilateral intracerebral bleeding and recovered without any neurological sequelae. However, there is no relevant research on risk factors of cerebral hemorrhage after heatstroke. To the best of our knowledge, this study is the first to show that Cre level after heatstroke is positively correlated with the risk of cerebral hemorrhage in multivariate analysis. Cerebral hemorrhage risk was also positively correlated with DIC scores, suggesting that DIC severity is positively correlated with cerebral hemorrhage. Cerebral hemorrhage was not associated with patients whether they had combined with DIC.

First, our study found that higher Cre levels may be associated with a higher risk of cerebral hemorrhage, and reviewing previous studies, we found that elevated Cre is associated with other causes of cerebral hemorrhage.^[14]^ It is more likely to play a causal role in the risk of ICH than ischemic stroke or is more linked to the mechanism of the disease. Some evidence suggests that the possible mechanism underlying this phenomenon involves the kidney and brain microvascular beds, which are composed of small, short vessels that arise from large arteries with high pressure (“strain vessels”); moreover, low arterial resistance may be involved, creating a vulnerability to other risk factors.^[15–17]^ The complications of renal impairment (anemia, acidosis, uremia, altered calcium-phosphate metabolism, and platelet dysfunction) may lead to vascular calcification, endothelial dysfunction,^[18]^ and decreased cerebral perfusion, promoting tissue hypoxia and worsening stroke outcome. Therefore, patients with an early heatstroke with severe renal injury or elevated Cre levels require timely interventions to help prevent cerebral hemorrhage.

Furthermore, severe DIC has always been a risk factor for systemic bleeding, including cerebral hemorrhage. Many patients experience DIC after a heatstroke. Studies have found that cerebral hemorrhage may be caused by DIC in patients with acute promyelocytic leukemia and other tumors. Previous reports of heatstroke cases with cerebral hemorrhage suggest that it may be associated with DIC.^[4,9]^ Our study found that the higher the ISTH score, the higher the incidence of heatstroke. These findings suggest that DIC should be addressed early to help improve outcomes of heatstroke patients and prevent related cerebral hemorrhage.

These findings may help clinicians recognize cerebral hemorrhage early. Our study found that cerebral hemorrhage after heatstroke can lead to a significant increase in the mortality risk. Moreover, the most common sites of cerebral hemorrhage include the subarachnoid space, frontal lobe, and cerebellum, indicating that these structures may be vulnerable to injury after heatstroke; these findings are consistent with those of some previous studies.^[2,3,6]^

This study has some limitations. Heatstroke is a rare event. This study was a retrospective clinical study with a small sample size. This study was based on heterogeneous data and may provide a reference for clinical diagnosis and treatment.

## 5. Conclusions

Heatstroke-related cerebral hemorrhage may be associated with elevated Cre levels and DIC severity (ISTH score) after heatstroke, and heatstroke with cerebral hemorrhage may accelerate death.

## Author contributions

**Conceptualization:** Guodong Lin.

**Methodology:** Guodong Lin, Xiaogan Hou.

**Project administration:** Guodong Lin.

**Resources:** Guodong Lin, Zhiguo Pan.

**Writing – original draft:** Guodong Lin, Chongxiao Xu, Hailun Peng.

**Data curation:** Jieyi Wu, Hailun Peng, Anwei Liu, Xuan He, Wenda Chen, Xiaogan Hou.

**Software:** Jieyi Wu.

**Visualization:** Hailun Peng.

**Formal analysis:** Anwei Liu.

**Supervision:** Qiang Wen, Zhiguo Pan.

**Validation:** Qiang Wen.

**Writing – review & editing:** Zhiguo Pan.
